# Disrupted Functional Connectivity Within and Between Resting-State Networks in the Subacute Stage of Post-stroke Aphasia

**DOI:** 10.3389/fnins.2021.746264

**Published:** 2021-12-01

**Authors:** Chao Zhang, Yingying Xia, Tao Feng, Ke Yu, Haiyan Zhang, Muhammad Umair Sami, Jie Xiang, Kai Xu

**Affiliations:** ^1^Department of Radiology, Affiliated Hospital of Xuzhou Medical University, Xuzhou, China; ^2^Department of Rehabilitation, Affiliated Hospital of Xuzhou Medical University, Xuzhou, China; ^3^Department of Radiology, The Second Affiliated Hospital of Xuzhou Medical University, Xuzhou, China

**Keywords:** resting-state, stroke, aphasia, independent component analysis, functional connectivity

## Abstract

**Background:** Post-stroke aphasia (PSA) results from brain network disorders caused by focal stroke lesions. However, it still remains largely unclear whether the impairment is present in intra- and internetwork functional connectivity (FC) within each resting-state network (RSN) and between RSNs in the subacute stage of PSA.

**Objectives:** This study aimed to investigate the resting-state FC within and between RSNs in patients with PSA and observe the relationships between FC alterations and Western Aphasia Battery (WAB) measures.

**Methods:** A total of 20 individuals with subacute PSA and 20 healthy controls (HCs) were recruited for functional MRI (fMRI) scanning, and only patients with PSA underwent WAB assessment. Independent component analysis was carried out to identify RSNs. Two-sample *t*-tests were used to calculate intra- and internetwork FC differences between patients with PSA and HCs. The results were corrected for multiple comparisons using the false discovery rate (FDR correction, *p* < 0.05). Partial correlation analysis was performed to observe the relationship between FC and WAB scores with age, gender, mean framewise displacement, and lesion volume as covariates (*p* < 0.05).

**Results:** Compared to HCs, patients with PSA showed a significant increase in intranetwork FC in the salience network (SN). For internetwork FC analysis, patients showed a significantly increased coupling between left frontoparietal network (lFPN) and SN and decreased coupling between lFPN and right frontoparietal network (rFPN) as well as between lFPN and posterior default mode network (pDMN) (FDR correction, *p* < 0.05). Finally, a significant positive correlation was found between the intergroup difference of FC (lFPN-rFPN) and auditory-verbal comprehension (*p* < 0.05).

**Conclusion:** Altered FC was revealed within and between multiple RSNs in patients with PSA at the subacute stage. Reduced FC between lFPN and rFPN was the key element participating in language destruction. These findings proved that PSA is a brain network disorder caused by focal lesions; besides, it may improve our understanding of the pathophysiological mechanisms of patients with PSA at the subacute stage.

## Introduction

Post-stroke aphasia (PSA) is a clinical syndrome originating from the damage of language ability and comprehension due to localized stroke lesions (Thiel and Zumbansen, 2016). Language function remodeling in PSA is characterized by temporal and spatial variability (Stefaniak et al., 2020). The reperfusion and inflammatory response of the perilesional tissue largely disappears in the subacute phase of PSA, and the brain is considered to be in a relatively more stable state than in the acute stage (Fu et al., 2015; Boyd et al., 2017); this is also an effective therapeutic window for speech rehabilitation (Brady et al., 2016; Fridriksson and Hillis, 2021). Therefore, understanding the pathophysiological mechanism of the subacute stage of PSA is very important for aphasia treatment, as the field moves from standardized therapies toward more targeted individualized treatment strategies (Thiel and Zumbansen, 2016).

The brain is a complex network composed of multiple subnetworks supporting different functional properties (Shirer et al., 2012). Resting-state functional MRI (rs-fMRI) is an established tool to explore intrinsic brain activity non-invasively and effectively, and it has been widely used in neuropsychiatric diseases (Liu et al., 2015, 2017; Zhang et al., 2020). Regional brain activity-based rs-fMRI analysis of patients with PSA (i.e., stroke occurred in the left hemisphere) showed decreased local synchronization in multiple brain areas of the left hemisphere (Yang et al., 2016). Functional connectivity (FC)-based rs-fMRI analysis has been used to evaluate the interaction between language network and the whole brain in patients with PSA, with impressive findings (Zhu et al., 2014). A study by Balaev et al. (2016) reported that FC in multiple subnetworks was demonstrated to be disrupted in patients with PSA, by using independent component analysis (ICA).

Previous studies summarized that PSA showed a three-phase model of dynamic functional organization (Nenert et al., 2018; Siegel et al., 2018; Stockert et al., 2020), such as decreased global brain activation in the acute phase, increased activation in bilateral domain-general networks and perilesional cortex during the subacute phase, and subsequent normalization of language-related areas in the chronic phase. It was also proved that focal stroke lesions can affect the functional organization of a certain subnetwork and disrupt the internetwork FC between these subnetworks (Wang C. et al., 2014). However, neither regional brain activity-based analysis nor FC-based analysis of the whole brain network can provide the complete picture of intra- and internetwork FC of the subnetworks in patients with PSA.

To our knowledge, no study so far has evaluated the intra- and internetwork FC of the whole brain to investigate the pathophysiological mechanisms of PSA at the subacute stage. As a multivariate data-driven analysis method, ICA can identify multiple resting-state networks (RSNs) and investigate both intra- and internetwork FC *in vivo* (Wang D. et al., 2014). ICA has been previously applied to analyze intra- and internetwork FC in many neurological and psychiatric disorders (Jafri et al., 2008; Song et al., 2013). We performed the ICA of rs-fMRI data to investigate the intra- and internetwork FC between patients with PSA at the subacute stage and healthy controls (HCs). It is hoped that this study can likely improve our understanding of the potential pathophysiological mechanism of PSA at the subacute stage.

## Materials and Methods

### Participants

**FIGURE 1:**
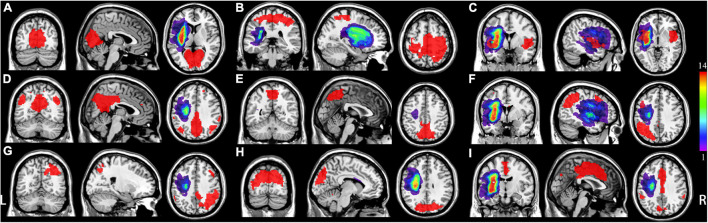
Distribution of each individual lesion for all patients with post-stroke aphasia (PSA). Colors represent the number of patients. L represents left, and R represents right. **(A)** Medial visual network (mVN), **(B)** sensorimotor network (SMN); **(C)** salience-related network (anterior insular cortex); **(D)** posterior default mode network (pDMN); **(E)** visual-spatial network (VisN); **(F)** left frontoparietal network (lFPN); **(G)** right frontoparietal network (rFPN); **(H)** lateral visual network (lVN); **(I)** salience-related network (anterior cingulate cortex).

A convenience sample of 20 hospitalized patients with PSA were recruited in this study. All patients were right-handed native speakers and were evaluated by MRI scanning and Western Aphasia Battery (WAB) testing 1 month after stroke. All stroke lesions were located in the left middle cerebral artery blood supply area (refer to Figure 1 for the lesion overlap map). None of the patients received language-related therapy. No severe cerebral white matter lesion was revealed through conventional MRI. The exclusion criteria were as follows: (i) MRI revealed other lesions in the brain parenchyma, such as intracranial hemangioma or tumor; (ii) history of head trauma or mental illness; and (iii) presence of metal in the body as a contraindication to MRI scanning. All patients underwent routine medical treatment, and none of them received any other relevant interventions. Of note, 20 healthy, age-, and gender-matched volunteers with right-handed were selected as HCs for this study. The inclusion criteria for HCs were as follows: (i) absence of any signs and symptoms of neurological disorders and (ii) no history of the long-term use of drugs that could affect the nervous system. The exclusion criteria were as follows: (i) neurological disorders or family history of genetic disorders and (ii) poor quality of acquired MRI images.

### MRI Data Acquisition

A 3.0 Tesla MRI scanner (GE Medical Systems, Signa HD, Waukesha, WI, United States) with an eight-channel head coil was used to scan all participants. To minimize head motion, the head of an individual was stabilized with comfortable foam pads, and all subjects wore earplugs to reduce the noise during MRI scanning. 3D-T1 BRAVO sequence was used to acquire a high-resolution T1-weighted image (T1WI) for the whole brain with a repetition time (TR) = 7 ms; echo time (TE) = 3 ms; field of view (FOV) = 256 mm × 256 mm; number of slices = 192; flip angle = 12°; and isotropic spatial resolution = 1 mm × 1 mm × 1 mm. Then, resting blood oxygen level-dependent images were acquired using an echo-planar imaging sequence. The scanning parameters were as follows: TR = 2000 ms; TE = 30 ms; FOV = 220 mm × 220 mm; slice thickness = 3 mm; slice gap = 1 mm; voxel size = 3.4 mm × 3.4 mm × 4.0 mm; number of slices = 36; flip angle = 90°; and total volume of each subject = 185.

### Lesion Mapping

The lesion of each patient was manually delineated by two radiologists (KY and YX) on individual high-resolution T1WI images using ITK-SNAP^1^ (Yushkevich et al., 2006; Figure 1). Thus, we obtained two values for each lesion volume, which were automatically generated by ITK-SNAP. Weighted kappa (κ) statistics were used to assess interobserver agreement for lesion delineation. Finally, each individual volume obtained from each radiologist was averaged as the final result for each patient.

### Resting-State Functional MRI Data Preprocessing

A graph theoretical network analysis toolbox for imaging connectomics (GRETNA)^2^ was used to perform data preprocessing (Wang et al., 2015). The steps used are outlined as follows: (i) the first 10 time points of each subject were removed; (ii) slice timing was used to correct time differences on the remaining 175 volumes; and (iii) realigning was used to correct individual-level head motion through a Friston-24 model, i.e., any subject with a head maximum displacement > 2 mm, maximum rotation >2.0°, or mean framewise displacement (FD) >0.3 was excluded from the study (Yan et al., 2013; Zhang et al., 2019). To further minimize the potential influences of head motion, mean FD was set as a covariate for further second-level statistics (Satterthwaite et al., 2012; Zeng et al., 2014). Subsequently, the individual structural images were segmented and normalized to the Montreal Neurological Institute (MNI) space by using a “clinical toolbox” in Statistical Parametric Mapping version 12 (SPM12) software^3^ implemented in MATLAB R2013b (MathWorks, Natick, MA, United States), which employed a cost-function modification to exclude the lesion area, avoiding bias during spatial normalization (Brett et al., 2001). This process has been widely used in other studies (Stebbins et al., 2008; Yang et al., 2018). The motion-corrected functional imaging data were normalized to MNI space using these transformation parameters and resampled to a voxel size of 3 mm × 3 mm × 3 mm. The functional volumes were spatially smoothed with Gaussian kernel 6-mm full-width-at-half-maximum.

### Independent Component Analysis

The preprocessed data of all subjects were put into one folder. GIFT software^4^ was employed to perform group spatial ICA (Calhoun et al., 2001). The procedures included the following steps: (i) data reduction was performed through principal component analysis; (ii) the number of independent components (ICs) was automatically estimated using the minimum description length; (iii) the InfoMax algorithm was utilized for the group analysis of ICA. To ensure the stability of estimation, this algorithm was repeated 100 times in ICASSO, and the most central run was selected and analyzed further; and (iv) back reconstruction for individual-level components (Erhardt et al., 2011). Each component for the maps of all participants was evaluated by a random-effect one-sample *t*-test using a family-wise error correction (*p* < 0.05) through SPM12 software implemented in MATLAB R2013b and an extent threshold of 50 voxels (Zhang et al., 2017).

## Statistical Analysis

A Chi-square test was employed to calculate the gender difference between the two groups, and the two-sample *t*-tests were used to observe the intergroup age difference. The internetwork FC differences between the two groups were calculated using the two-sample *t*-tests, and results were corrected for multiple comparisons using the FDR correction (*p* < 0.05). The intergroup differences in the intranetwork FC were compared in a voxel-wise manner using two-sample *t*-tests with masks generated from the results of IC one-sample *t*-tests (FDR correction, *p* < 0.05, cluster size of at least 30 voxels). To understand whether significant differences of intra- or internetwork FC can contribute to the symptoms of aphasia (using WAB tests), partial correlation analyses were performed to observe the relationship between FC and WAB scores with age, gender, mean FD, and lesion volume as covariates (*p* < 0.05).

## Results

### Demographic and Clinical Data

The details of age, gender, and WAB score for each patient are listed in Table 1. The results showed no significant difference in age (*p* = 0.74) and gender (*p* = 0.74) between the patients with PSA and HCs. High interobserver agreement (weighted κ = 0.80) was found for lesion delineation. The mean lesion volume of patients was 32.00 ± 14.08 cm^3^ (Supplementary Table 1).

**TABLE 1:** Demographics and clinical data.

**Variable**	**PSA (*N* = 20)**	**HCs (*N* = 20)**	***p***
Gender (M/F)	11/9	7/13	0.74^#^
Age (years)	46.5 ± 11.9	45.3 ± 13.5	0.74*
WAB measures			
Yes/no questions	50.36 ± 3.88	/	/
Word recognition	44.54 ± 7.47	/	/
Sequential commands	47 ± 17.27	/	/
Repetition	65.73 ± 12.67	/	/
Object naming	26.9 ± 12.84	/	/
Word fluency	2.18 ± 2.6	/	/
Sentence completion	5.81 ± 3.95	/	/
Responsive speech	5.45 ± 2.87	/	/
Information content	5 ± 2.83	/	/
Fluency	6.18 ± 1.6	/	/

*PSA, post-stroke aphasia; HCs, healthy controls; M, male; F, female; WAB, Western Aphasia Battery.*

*Data are presented as the range and mean ± SD.*

*^#^The *p*-value was obtained using a chi-square test.*

*The *p*-value was obtained by a two-sample *t*-test.

### Networks of Interests

**FIGURE 2:**
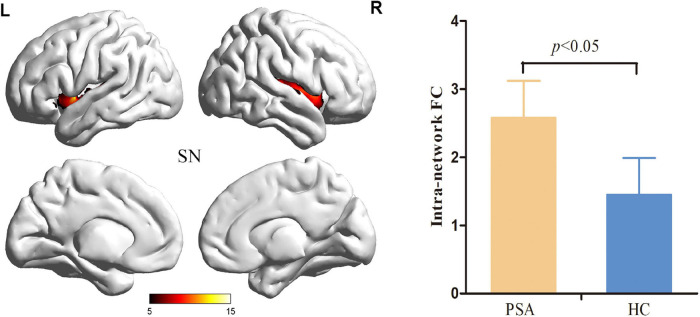
Salience Network with significant changes in intranetwork FC in patients with PSA. SN, salience network; FC, functional connectivity; PSA, post-stroke aphasia.

From a total of 23 ICs, 9 ICs were judged to represent RSNs (Figure 2), such as medial visual network (mVN), sensorimotor network (SMN), posterior default mode network (pDMN), anterior insular cortex of salience network (SN1), visuospatial network (VisN), left frontoparietal network (lFPN), right frontoparietal network (rFPN), lateral visual network (lVN), and anterior cingulate cortex of salience network (SN2) (Shirer et al., 2012; Ma et al., 2016; Zhang et al., 2017, 2018).

### Differences Between Groups in Intra- and Internetwork Functional Connectivity

**FIGURE 3:**
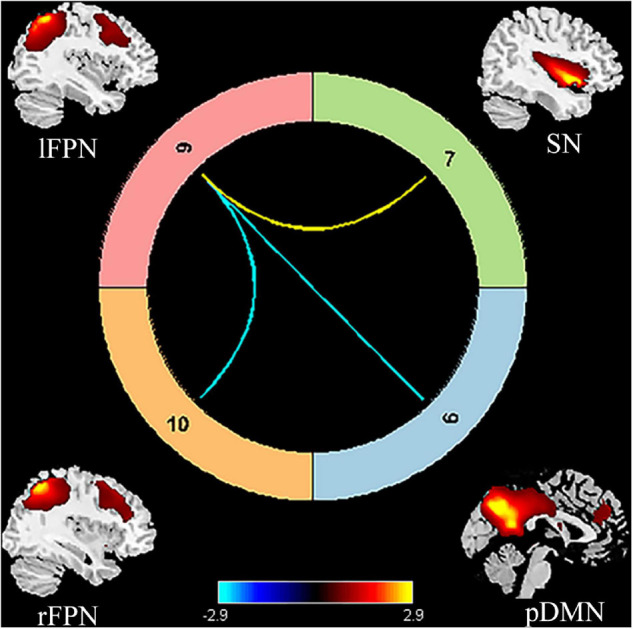
Significant internetwork FC differences between patients with PSA and HCs. The yellow line represents significantly increased internetwork FC, and the blue lines denote significantly decreased internetwork FC in patients with PSA when compared to HCs. FC, functional connectivity; PSA, post-stroke aphasia; HCs, healthy controls; pDMN, posterior default mode network; SN, salience network; lFPN, left frontoparietal network; rFPN, right frontoparietal network.

**FIGURE 4:**
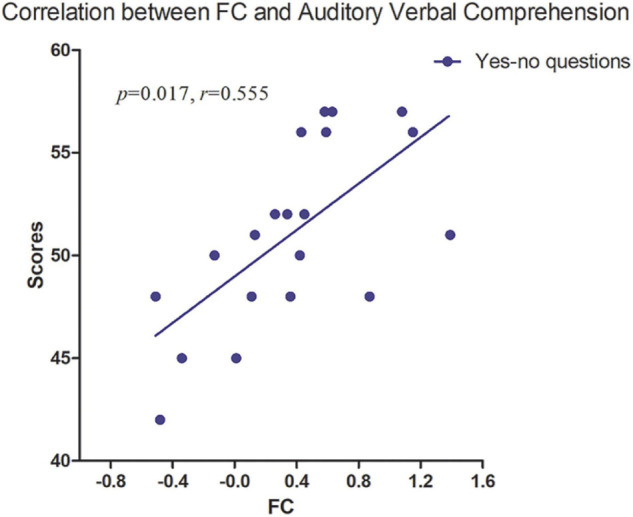
The strength of FC between lFPN and rFPN was positively correlated with Yes/No questions (*p* = 0.017, *r* = 0.555). FC, functional connectivity; lFPN, left frontoparietal network; rFPN, right frontoparietal network.

Compared to HCs, patients with PSA showed significantly increased intranetwork FC in the SN1 (Figure 3) (FDR correction, *p* < 0.05). For internetwork FC analysis, patients showed a significantly increased coupling between lFPN and SN1 and a decreased coupling between lFPN and rFPN as well as between lFPN and pDMN (FDR correction, *p* < 0.05) (Figure 4).

No significant correlation was found between intranetwork FC and WAB tests. Partial correlation analysis revealed a significant positive correlation between intergroup difference of FC (lFPN with rFPN) and auditory-verbal comprehension (Yes/No questions) with age, gender, FD, and lesion volume as covariates (*p* < 0.05) (Figure 4), which was performed in SPSS version 16 (SPSS Inc., Chicago, IL, United States).

## Discussion

In this study, we carried out ICA and followed by intra- and internetwork analysis to compare resting-state spontaneous brain activity in patients with PSA at the subacute stage and the HCs. We found the following results: (i) significant differences in intra- and internetwork FC between patients with PSA and HCs; (ii) decreased coupling between lFPN and rFPN was significantly correlated with auditory-verbal comprehension of WAB; and (iii) no significant relationship was noted between intranetwork FC and WAB assessment.

### Altered Functional Connectivity Within Resting-State Network

We found that the intranetwork FC of SN was enhanced in patients with PSA when compared to HCs. The SN, which encompasses the bilateral anterior insular cortex and the anterior cingulate cortex, is responsible for evaluating surrounding information and internal events to switch the relevant processing system (Uddin, 2015). In our study, altered intranetwork FC was located in a part of the SN, i.e., bilateral insular cortex, which has been proved to be an important structure in language production (Uddin et al., 2017). Moreover, fMRI studies proved that language and reading skills were closely correlated with both intra- and internetwork synchronization of the anterior insular cortex (Supekar and Menon, 2012; Zaccarella and Friederici, 2015; Chang et al., 2018). The increased intranetwork of the bilateral anterior insular cortex, especially the left anterior insular cortex near the stroke lesion that still showed an increased activation, indicated a compensatory reorganization for aphasia at the subacute stage.

### Altered Functional Connectivity Between Resting-State Network

We found that pDMN and rFPN showed a decreased coupling with lFPN; in contrast, SN presented an increased FC with lFPN. As an asymmetrical network, the FPN was divided into lFPN and rFPN. The lFPN included the classical language-related brain areas, such as Broca’s area and Wernicke’s area (Harding et al., 2015). The lFPN identified in our study covered the left middle frontal lobe, left supramarginal gyrus, angular gyrus, and left precuneus, which broadly include language ability-related regions. The brain structure of rFPN was mainly composed of the right angular gyrus, right dorsolateral prefrontal cortex, right frontal oculomotor area, and right precuneus (Balaev et al., 2016). Language dysfunction has been proved to be closely related to intrinsic lFPN connectivity through a static rs-fMRI study (Vlooswijk et al., 2010; Zhu et al., 2014). We found patients with PSA showing a significant decrease in FC between lFPN and rFPN. This may be due to the distribution of lFPN regions that were mainly located in the blood supply area of the left middle cerebral artery, where the stroke lesions occurred in this study. Furthermore, it was not an accidental finding that pDMN and rFPN showed abnormal coupling with lFPN. Compared with previous reports, we have newly found the coupling interruption between the bilateral FPN in patients with PSA at the subacute stage. In addition, we found that the auditory-verbal comprehension disability on WAB tests was positively correlated with decreased FC between the lFPN and right cerebral hemisphere, which is consistent with previous literature reports (Zhu et al., 2014; Balaev et al., 2016). These findings indicated that focal stroke lesions of the left hemisphere influenced the language comprehension ability by disrupting the brain network coupling in PSA at the subacute stage.

The concept of DMN, defined as a baseline state of the normal adult human brain, was identified by Raichle et al. (2001) through PET and fMRI. DMN was further described as two parts, namely, (i) the medial prefrontal cortex and anterior cingulate were classified as the anterior DMN (aDMN) and (ii) posterior cingulate cortex (PCC), precuneus, and bilateral angular gyrus were classified as the pDMN (Wang C. et al., 2014; Zhang et al., 2017). As an important cortical center with high metabolic activity, pDMN had connections to a wide range of brain regions (Greicius et al., 2003), and it was also shown to be primarily responsible for connections to the FPN (Leech et al., 2012). Many studies found that pDMN was tightly connected to the key region in language processing within FPN (Crone et al., 2006; Lambon Ralph, 2010). Our study showed reduced coupling between lFPN and pDMN in patients with PSA, which further validated the previous view.

The DMN has been proven to be closely correlated with cognitive function in healthy individuals (Mak et al., 2017), as well as some neurological diseases, such as epilepsy (Zhang et al., 2017), Alzheimer’s disease (Dennis and Thompson, 2014), and Parkinson’s disease (Rektorova, 2014). DMN, FPN, and SN constitute the “triple network model” of aberrant saliency mapping and cognitive dysfunction in psychopathology (Menon, 2011). The “triple network model” has been reported to be involved in autism, cognition impairment, schizophrenia, and obsessive-compulsive disorder (Menon, 2011; Gürsel et al., 2020). SN has a pivotal role in switching between DMN and FPN, which represents shifting between self-referential thoughts (modulated by the DMN) and goal-directed behavior (modulated by the FPN) (Seeley et al., 2007; Dodds et al., 2011). Few studies report the impairment of the “triple network model” in patients with PSA. Abnormal FC of pDMN/lFPN and SN/lFPN indicated a disrupted regulation in the “triple network model” in this study. However, no significant correlation was found between the strength of the “triple network model” coupling and WAB score. Our findings demonstrated that the destruction of the “triple network model” was revealed in the subacute phase of PSA either, but further study is needed to verify the correlation with language ability.

The current results showed differences from previous studies. These discrepancies may be related to that the time point of the recruitment of a patient was different. Patients in other studies were selected 1 day or 1–10 months after stroke, whereas our patients had a shorter time span (typically at 1 month after stroke), which means our patients have temporal homogeneity.

### Limitations

There were some limitations that should be noted in our study. First, all patients were on conventional medication. Although patients underwent fMRI scanning while they were off medication for at least 24 h, and the effects of treatment could not be completely ruled out. Second, the current sample size was relatively small, and a larger sample size might be better for further analysis. Third, only patients at the subacute stage were analyzed because the neurobiological state of the brain was relatively stable in this phase, such as less reperfusion of brain tissue or cell regeneration than at the acute stage (Stefaniak et al., 2020). Fourth, it cannot be ruled out that intranetwork FC of the left anterior insular cortex of SN (2 cases of 20) may be affected by cerebrovascular changes due to which was adjacent to the lesion. It would be better if the future study can verify whether there is an influence between cerebrovascular changes and cortical network dysfunction. Finally, the head motion effect may not be fully ruled out although we implemented a series of procedures to reduce the impact of head movement.

## Conclusion

We found the abnormalities of FC within and between multiple brain networks in patients with PSA at the subacute stage. Reduced lFPN-rFPN connectivity was closely associated with Yes/No questions, indicating that brain focal lesions influence language comprehension ability by disrupting the brain network. Revealing the patterns of the brain network FC may improve our understanding of the pathophysiological mechanisms and may also contribute to clinical therapy.

## Data Availability Statement

The original contributions presented in the study are included in the article/Supplementary Material, further inquiries can be directed to the corresponding author/s.

## Ethics Statement

The studies involving human participants were reviewed and approved by Xuzhou Affiliated Hospital, Xuzhou Medical University. The patients/participants provided their written informed consent to participate in this study.

## Author Contributions

CZ, TF, JX, and KX contributed to the research project conception. CZ and TF contributed to the research project organization and execution. CZ, KY, and YX contributed to statistical analysis, design, and execution. HZ and KX contributed to the review of the statistical analysis. CZ, HZ, and MS contributed to the writing of the first draft. TF, JX, and KX contributed to the review of the manuscript. CZ, JX, and KX took responsibility for the data. All authors contributed to the article and approved the submitted version.

## Conflict of Interest

The authors declare that the research was conducted in the absence of any commercial or financial relationships that could be construed as a potential conflict of interest.

## Publisher’s Note

All claims expressed in this article are solely those of the authors and do not necessarily represent those of their affiliated organizations, or those of the publisher, the editors and the reviewers. Any product that may be evaluated in this article, or claim that may be made by its manufacturer, is not guaranteed or endorsed by the publisher.

## Footnotes

1
http://www.itksnap.org

2
https://www.nitrc.org/projects/gretna/

3
http://www.fil.ion.ucl.ac.uk/spm/

4
https://trendscenter.org/software/gift/
