# Supplementary material for: Disrupted Functional Connectivity Within and Between Resting-State Networks in the Subacute Stage of Post-stroke Aphasia
Source: Front Neurosci. 2021 Dec 1;15:746264. doi: 10.3389/fnins.2021.746264 (PMC8672309; doi:10.3389/fnins.2021.746264)
Supplement: Supplementary file 1 [file Data_Sheet_1.pdf]

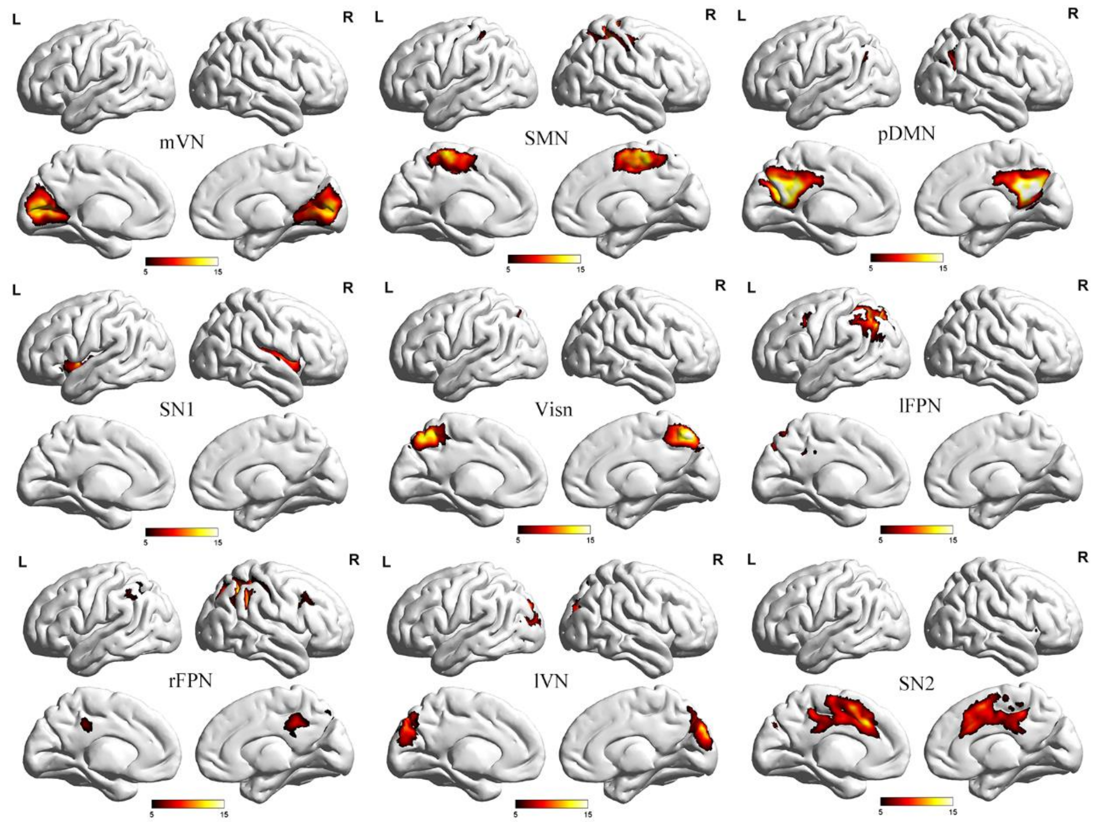

Figure S1. Three-dimensional images made by BrainNet Viewer. Nine RSNs identified by GIG-ICA across all subjects. Functional maps are shown on the lateral and medial surfaces of bilateral hemisphere. The color scale represents the t values in each RSN. Abbreviations: mVN, medial visual network; SMN, sensorimotor network; pDMN, posterior default-mode network; SN1, salience related network 1 (anterior insular cortex); Visn, Visual spatial network; lFPN, left frontoparietal network; rFPN, right frontoparietal network; lVN, lateral visual network; SN2, salience related network 2 (anterior cingulate cortex).

Table S1 Lesion volume of each patient with PSA

Number	Sex	Age	Lesion Volume (cm^3)
P003	M	47	28.32
P004	M	43	21.11
P005	M	46	43.32
P006	F	69	24.82
P008	F	32	57.03
P009	F	51	43.35
P010	F	54	24.76
P011	M	38	51.52
P012	F	50	42.90
P013	M	31	25.69
P014	M	66	50.35
P015	M	47	17.31
P018	M	33	42.91
P019	M	62	8.98
P020	F	65	7.41
P021	M	50	25.33
P022	F	33	32.05
P023	F	43	28.37
P024	M	39	21.12
P025	F	34	43.36

M, male; F, female; PSA, post-stroke aphasia.
